# Group 3 ILCs: Peacekeepers or Troublemakers? What's Your Gut Telling You?!

**DOI:** 10.3389/fimmu.2019.00676

**Published:** 2019-04-05

**Authors:** Eirini Pantazi, Nick Powell

**Affiliations:** ^1^Kennedy Institute of Rheumatology, University of Oxford, Oxford, United Kingdom; ^2^Department of Inflammation Biology, Centre for Inflammation and Cancer Immunology, King's College London, London, United Kingdom

**Keywords:** group 3 innate lymphoid cells, symbiosis, intestinal inflammation, IBD, Crohn's disease, ulcerative colitis

## Abstract

A complex network of interactions exists between the microbiome, the epithelium, and immune cells that reside along the walls of the gastrointestinal tract. The intestinal immune system has been assigned with the difficult task of discriminating between commensal, harmless bacteria, and invading pathogens that translocate across the epithelial monolayer. Importantly, it is trained to maintain tolerance against commensals, and initiate protective immune responses against pathogens to secure intestinal homeostasis. Breakdown of this fine balance between the host and its intestinal microbiota can lead to intestinal inflammation and subsequently to development of inflammatory bowel disease (IBD). A decade since their discovery, innate lymphoid cells (ILCs) are now recognized as important regulators of intestinal homeostasis. ILC3s have emerged as a critical subset in the gut. They are the most phenotypically diverse ILC population and interact directly with numerous different cell types (haematopoietic and non-haematopoeitic), as well as interface with the bacterial flora. In addition to their contribution to intestinal pathogen immunity, they also mitigate against tissue damage occurring following acute injury, by facilitating tissue repair and regeneration, a key function in the maintenance of intestinal homeostasis. However, in chronic inflammation the tables are turned and ILC3s may acquire a pro-inflammatory phenotype in the gut. Chronic ILC activation can lead to persistent inflammation contributing to IBD and/or colorectal cancer. In this review, we discuss current knowledge of group 3 ILCs and their contributions to intestinal homeostasis and disease leading to novel therapeutic targets and clinical approaches that may inform novel treatment strategies for immune-mediated disorders, including IBD.

## Introduction

With more than 10^13^ microorganisms residing in the human gastrointestinal tract (1), our mucosal immune system has excelled in preserving intestinal homeostasis by generating protective immune responses against invading, harmful pathogens whilst maintaining tolerance toward commensals. However, breakdown of this fine balance may lead to excessive immune activation, persistent inflammation and ultimately to the development of inflammatory bowel diseases (IBD). The most common IBD phenotypes, Crohn's disease (CD) and ulcerative colitis (UC) are characterized by alternating phases of clinical relapse and remission. In CD inflammation can occur in any part of the gastrointestinal tract, whereas in UC inflammation is restricted to the colon (2). Despite the increasing incidence of IBD in the Western world (3), its complex etiology is yet to be fully understood. In general, it is thought that IBD is caused by a dysregulated immune response against the commensal bacterial flora in a genetically predisposed host (4). In accordance with this notion, genome-wide association studies (GWAS) have so far associated single nucleotide polymorphisms (SNPs) in more than 200 genetic loci with IBD susceptibility (5) including genes involved in bacterial recognition, epithelial barrier integrity and immune activation (6) highlighting the importance of microbiota-host interactions and the role of the intestinal immune system, in particular the innate one, in IBD pathogenesis.

### Innate Lymphoid Cells—A New Recruit to Mucosal Sentinel Duty

Defense against intestinal pathogens is multifaceted. The intestinal epithelium comprises a physical barrier, which together with the mucus layer and production of anti-microbial peptides provides a containment barrier, which confines microbes in the lumen. Cells of the innate immune compartment residing in the lamina propria are key early warning sentinels detecting invading pathogens through conserved pattern recognition receptors, such as toll-like receptors. Pathogen detecting populations, include cells of the mononuclear phagocyte system, including macrophages and dendritic cells (DCs), which engulf and process microbial antigens and then shape adaptive immune responses by providing initial signals to adaptive lymphocytes to engage potent antigen-specific T and B cell responses. Innate lymphoid cells (ILCs) are recent additions to the innate immune cell family (7–9). They are distributed throughout the human body in lymphoid and non-lymphoid tissues, but are especially enriched at the mucosal barrier surfaces (7), where they directly interact with a number of different cell types; hematopoietic or other (10–12). ILCs have lymphoid-like morphology, but lack any antigen-specific receptors. Arising from a common lymphoid progenitor and similarly to T cells, they can be further subdivided into phenotypically and functionally distinct populations that produce different combinations of effector cytokines to mediate their functions (9–11). Their development depends on different transcription factors, which are also used to help divide ILCs into 3 main groups; group 1 ILCs that includes the well characterized NK cells, as well as the non-cytotoxic ILC1s, group 2 ILCs or ILC2s, and finally group 3 ILCs including ILC3s and lymphoid tissue inducer (LTi) cells. However, recently discovered regulatory ILCs or ILCregs (13) may now represent an additional, distinct ILC family member generating a potential new 4th group of ILCs.

**Figure 1:**
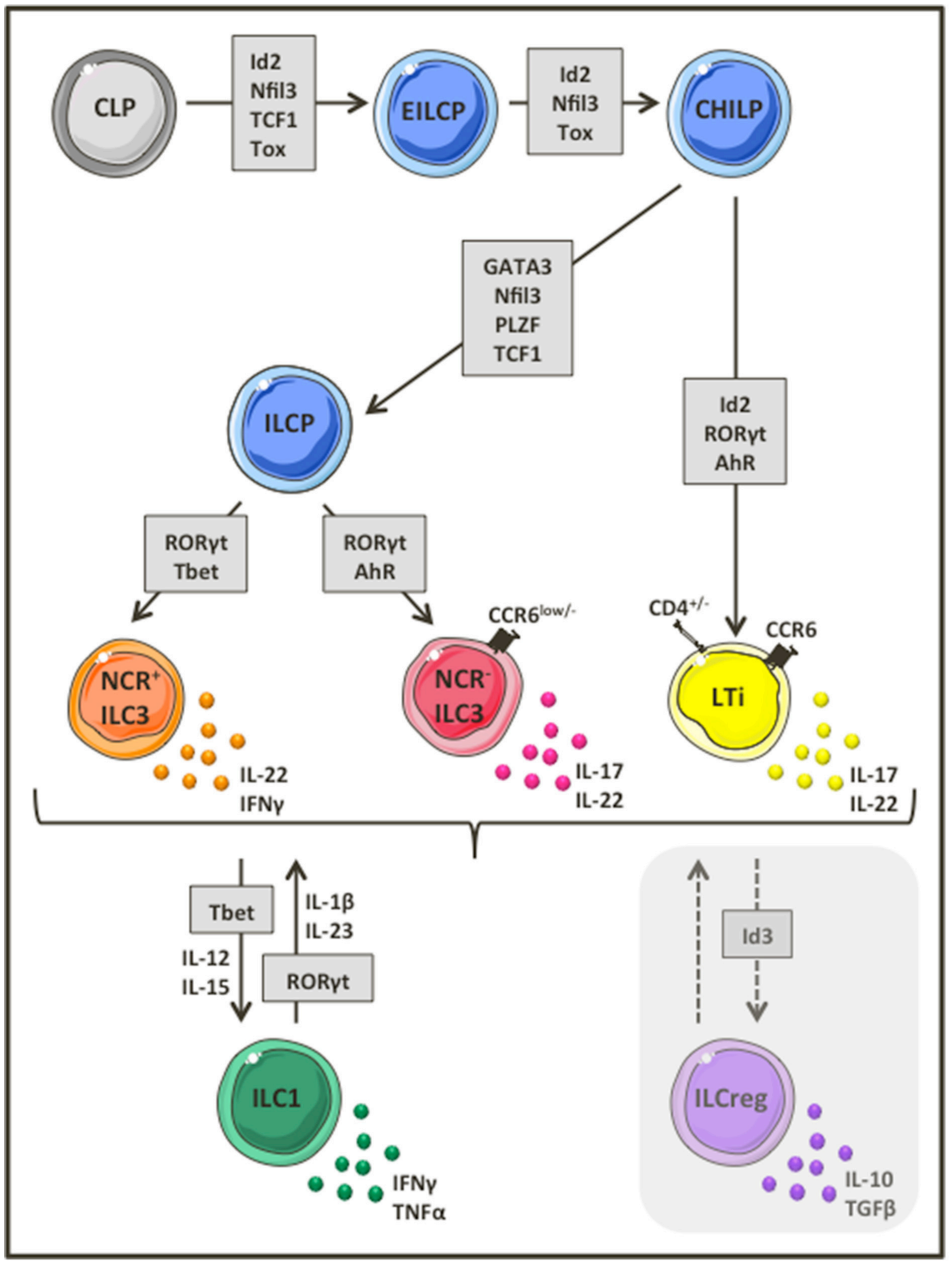
Group 3 ILC development and heterogeneity. Group 3 ILCs represent the most diverse ILC subset, comprising of CCR6^+^ LTi cells, and ILC3s that can be further divided into NCR^−^ and NCR^+^ ILC3s. Group 3 ILCs rise from a common lymphoid progenitor that lost T, B and eventually NK cell potential and depend on the transcriptional factor RORγt for their development. Depending on the environmental stimulus, ILC3s produce a variety of distinct cytokines such as IL-17, IL-22, IFNγ, and GM-CSF to mediate their functions. Interestingly, ILC3s are highly plastic cells and as such NCR^−^ ILC3s can acquire NCR expression and became NCR^+^ ILC3s in the presence of a T-bet gradient or loose completely RORγt expression to differentiate into ILC1 like ILCs and vice versa.

Much of the early work describing ILCs focused on their developmental requirements and capacity for plasticity. An early ILC progenitor (EILCP) rising form a common lymphoid progenitor, which has lost T and B potential, gives rise to NK cells, as well as all ILC lineages (14–16). Downstream of EILCP, Id2 expressing common helper-like ILC precursor (CHILP) gives rise to all ILCs, but not to NK cells (14), whereas all ILCs (except lymphoid tissue inducer cells, LTis), arise from an ILC precursor (ILCP) that expresses both Id2, and PLZF (17) (Figure 1). Group 3 ILCs that represent the most diverse and possibly best-characterized ILC populations both in humans and mice (12), require RORγt for their development (18–20), while the transcriptional factor aryl hydrocarbon receptor (AhR) is essential for their maintenance (21, 22). In general, group 3 ILCs, can be further divided into CCR6^+^ LTi cells that may or may not express CD4, and CCR6^low/−^ ILC3s. Based on whether or not CCR6^low/−^ ILC3s express natural cytotoxicity receptors (NCRs), they are additionally subdivided into NCR^+^ and NCR^−^ ILC3s (Figure 1). Upon activation, NCR^−^ ILC3s and LTi cells produce mostly interleukin (IL)-17A, IL-17F and IL-22, while NCR^+^ ILC3s produce mainly IL-22 (20, 23–27). Several independent studies have provided evidence suggesting plasticity within group 3 ILCs (28, 29). NCR^−^ ILC3s can differentiate into NCR^+^ ILC3s in the presence of increasing expression of T-bet (30, 31), or even acquire an ILC1-like phenotype by completely losing RORγt expression (29, 32). Similarly, ILC1-like ILC3s may also differentiate *in vivo* into NCR^−^ ILC3s in the presence of a RORγt gradient (32, 33) (Figure 1). Given the enriched numbers of group 3 ILCs in the gut mucosa and their role in preserving intestinal homeostasis, additional studies are required to clarify whether this profound, finely tuned plasticity within group 3 ILCs is a mechanism to regulate intestinal inflammation, and whether this plasticity may also be extended to recently identified ILCregs (13).

Although group 3 ILCs are primarily known for their role in anti-bacterial immunity (20, 27), they emerge as key effector cells at barrier surfaces. Recent studies describe their involvement in several immune-mediated diseases such as psoriasis (34, 35), multiple sclerosis (36), or cancer (37, 38), improving our understanding of these highly plastic cells in controlling inflammation, while suggesting new ways of therapeutic immune intervention. In this review, we focus on the role of group 3 ILCs in the gut, during intestinal homeostasis and disease.

## Group 3 ILCs Promote Intestinal Peace

### Microbiota-ILC3 Interactions—A Key Partnership for a Finely Tuned Intestinal Immune System

**Figure 2:**
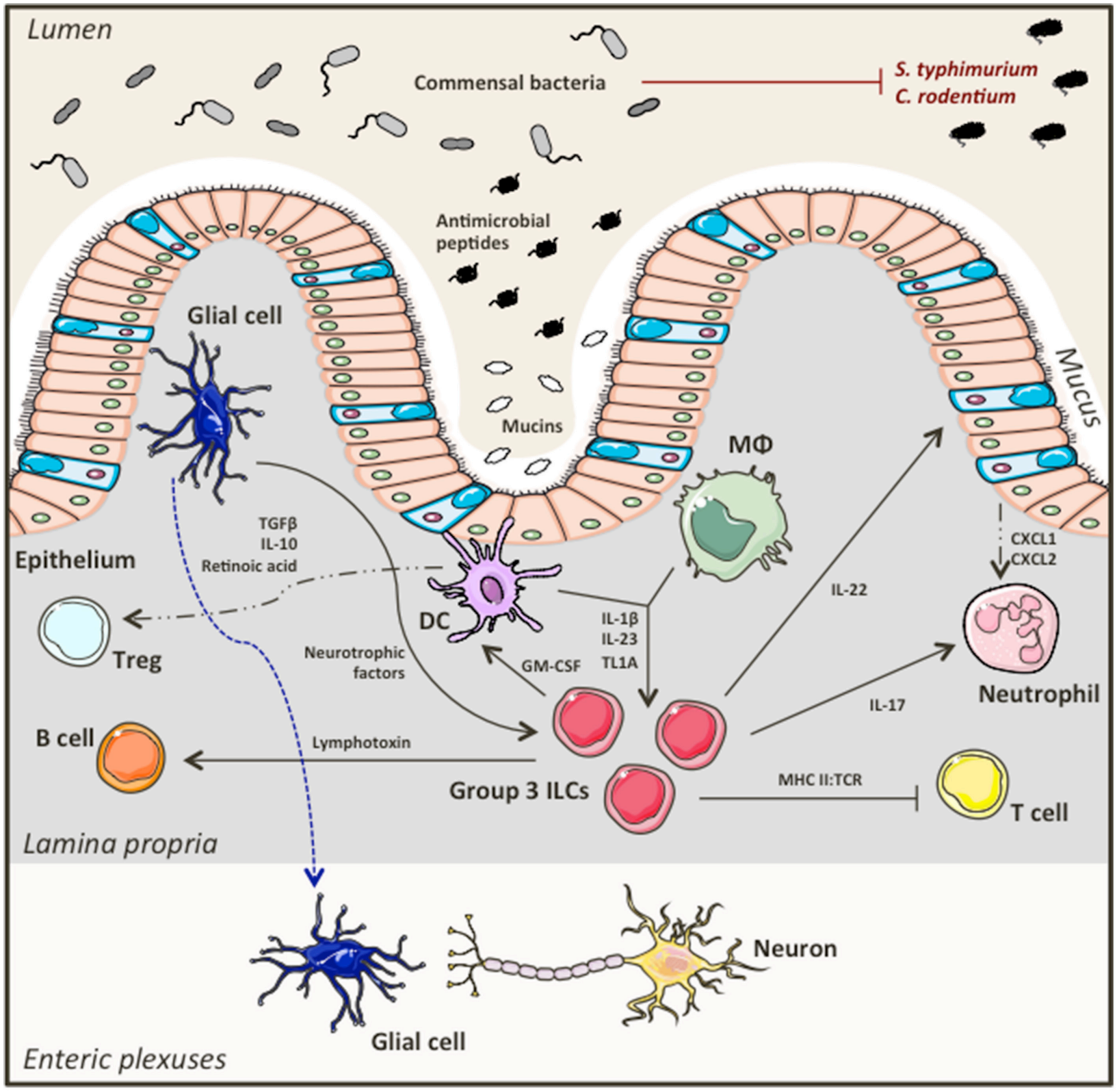
Group 3 ILCs in intestinal homeostasis. Group 3 ILCs including ILC3s and LTi cells directly interact with the commensal bacteria in the lumen, the intestinal epithelium, as well as with other immune cells and neurons in the lamina propria forming a finely tuned network that secures and maintains intestinal homeostasis.

Group 3 ILCs accumulate in the gastrointestinal tract and gut-associated lymphoid tissues in a microbiota-independent manner (21, 39–41). They directly interact with the bacterial flora, as well as with immune and non-hematopoietic cells creating a dynamic network between the host and its resident microbiota that favors symbiosis and preserves intestinal homeostasis (Figure 2). An important aspect of this partnership is containment of commensals to the lumen allowing for controlled bacterial sampling by lamina propria mononuclear phagocytes. ILC3s are key regulators of this process. Loss of ILC3s in the intestine leads to diminished IL-22 production (a key cytokine produced by ILC3s), and impaired production of antimicrobial peptides by intestinal epithelial cells, culminating in peripheral dissemination of *Alcaligenes* bacteria and systemic inflammation that could be prevented by exogenous replacement of IL-22 (41). ILC3s also play a key role in shaping adaptive immunity. Importantly, Hepworth et al. showed for the first time that loss of RORγt^+^ ILCs in immunocompetent mice lead to dysregulated adaptive immune responses against commensals, an effect that was not mediated by known ILC3 associated cytokines such as IL-17A, IL-22 and IL-23, but through MHC II:TCR interactions instead (42). In particular, selective deletion of MHC II expression on RORγt^+^ ILCs resulted in enhanced antigen-specific Th17 responses against bacterial flora, promoting spontaneous intestinal inflammation (42). Others have also reported contribution of ILC3s in the generation of Th17 responses against commensals (43), while antigen presenting capacities through MHC II and expression of co-stimulatory molecules have also been described for splenic ILC3s using *in vitro* systems of T cell priming (44). Although ILC3s can uptake, process and present antigen, they don't express co-stimulatory molecules and as such they can potentially result in T cell energy. Thus, when absent there is uncontrolled T cell activation and exaggerated T cell responses to commensals that would otherwise be regulated. Establishing a balanced, two-way relationship between ILC3s and T cells in the gut, T cells have proven to be crucial for keeping ILC3 numbers and functions in check. Absence of CD4^+^ T cells resulted in elevated ILC numbers, increased IL-22 production and subsequently enhanced secretion of anti-microbial peptides by epithelial cells (45). Other more complex, dynamic interactions between ILC3s, T cells and the microbiota are important at different development stages of the host. While ILC3s have an important role in influencing bacterial composition at early developmental stages, most likely to prevent unnecessary inflammatory responses, as bacteria burden expands, CD4^+^ T cells accumulate to establish tolerance using distinct mechanisms compared to their innate counterparts, that may become quiescent (46).

Although IL-22 plays a central role regulating mucosal immunity, the microbiota also play an important role fine-tuning the ILC3/IL-22 axis. Segmented filamentous bacteria (SFB) are commensal bacteria that selectively colonize the terminal ileum of mice, a key inductive site of mucosal immunity. They play a central role in the differentiation of Th17 cells (47, 48), but also regulate innate IL-22 production. Mono-association of germ free mice with SFB results in marked augmentation of IL-22 production by intestinal ILC3s. In this system IL-22 induced production of serum amyloid A proteins 1 and 2 from the intestinal epithelial cells, which in turn played a key role promoting the differentiation of Th17 cells (49). Other commensal bacteria may have an opposing role on IL-22 production, through induction of IL-25 by intestinal epithelial cells, which suppresses IL-22 production by RORγt^+^ ILCs (50).

The microbiota ILC3 partnership extends its branches to B cells too, the other major adaptive immune cell. In 2008, Tsuji et al. showed that LTi cells are essential for the formation of isolated lymphoid follicles (ILFs) and T cell independent generation of immunoglobulin A (IgA) by B cells in the gut (51). Later that year, a study published in Nature complemented these findings by showing that peptidoglycans from Gram^−^ bacteria activate LTi cells in the gut, which then induce chemokine production by stromal cells resulting in B cell recruitment and subsequently formation of ILFs (52). Kruglov et al. showed that RORγt^+^ ILCs mediate both T cell dependent and independent IgA production by B cells through secretion of soluble lymphotoxin α (sLTα3) and membrane-bound lymphotoxin β (LTα1β2), respectively (53). Beneficial interactions have also been described between group 3 ILCs and mononuclear phagocytes (54). Bacterial sampling by intestinal macrophages and DCs induces IL-1β secretion that activates ILC3s, which in turn produce GM-CSF that feeds back to mononuclear phagocytes to produce anti-inflammatory mediators such as IL-10 and retinoic acid eventually leading to Treg expansion and immune tolerance (54). Surprisingly, Ibiza et al. showed that group 3 ILCs also interact with the enteric nervous system in an attempt to maintain intestinal homeostasis (55). Interestingly, it was shown that glial cells in the lamina propria sense bacterial presence and secrete neurotrophic factors that induce IL-22 production by ILC3s through the neuroregulatory receptor RET (55), providing the first evidence of direct neuron involvement in innate immune regulation in the gut.

### ILC3s, Important Soldiers Fighting Foreign Pathogens

In accordance with studies showing how group 3 ILCs directly interact with the bacterial flora while working closely with other hematopoietic and non-immune cells to secure and maintain intestinal homeostasis, ILC3s have also been described as key effector cells in immunity against pathogens. Even prior to acquiring their official name, group 3 ILCs were associated with protection against *Proteobacteria* (20). Loss of NKp46^+^ RORγt^+^ IL-22 producing innate immune cells is linked to increased susceptibility to *Citrobacter rodentium* infection, which is a model for enteropathic *E.coli* infection (20) ILC3s were especially important as early producers of IL-22 (56). Lymphotoxin produced by RORγt expressing innate immune cells was necessary to control *C. rodentium* infection as lymphotoxin acted on the intestinal epithelium inducing CXCL1 and CXCL2 chemokine production and subsequently neutrophil recruitment at the early stages of infection (57). Sonnenberg et al. showed that infection with *C. rodentium* induced IL-23 mediated IL-22 production by CD4^+^ LTi cells, and that this subset of ILC3 was sufficient to promote immunity in immunodeficient hosts (27). More recent studies showed that expression of the G-protein-coupled receptor 183 (GPR183) on LTi cells is not only essential for their migration to cryptopatches and ILFs (58), but also required for ILC3-mediated protection against *C. rodentium* infection (59). Notably, immunity to *Citrobacter* is STAT3 dependent. STAT3 deficiency was associated with impaired IL-22 production, and increased disease severity, which could be rescued with exogenous IL-22 (60). STAT3 expression was only required in ILC3s and T cells to induce protection (60).

One of the mechanisms of IL-22 mediated regulation of the microbiota is through regulation of the glycosylation pattern of epithelial cells. IL-22 and lymphotoxin expressed by ILC3s control the expression of fucosyltransferase 2 (Fut2), which triggers fucosylation of epithelial cells, which in turn can be utilized as a nutrient source by luminal commensals (61). IL-22 mediated fucosylation of the epithelium was dependent on colonization of the GI tract with bacteria, and in a positive circuit, fucosylation promoted colonization with symbiotic bacteria, presumably by providing a favorable nutrient supply. Disruption of this system resulted in loss of host-microbe mutualism and rendered the host susceptible to *Salmonella typhimurium* infection (61). In accordance with these findings, Pickard et al. showed that IL-22 induced fucosylation is mediated by ILC3s upon activation with IL-23 produced by DCs during pathogen-induced stress (62). In this setting, rapid fucosylation could also improve tolerance to *C. rodentium* infection (62).

Klose et al. demonstrated that Tbet expressing; IFNγ producing RORγt^+^ ILCs are key drivers of immunity against *Salmonella* infections (30). Other work supports a role for ILC3s in protective immunity against *E. coli*, or *Klebsiella pneumoniae and Toxoplasma gondi* infection (63, 64). ILC3s also contribute to host resistance to Rotavirus. ILC3 derived IL-22 synergizes with IFNλ to minimize viral replication (65).

These studies point to an important role of group 3 ILCs in promoting symbiosis with commensals and protection against pathogens. ILC3s form a dynamic network of interactions with the microbiome, other immune cells, the intestinal epithelium and surprisingly with neuro-glial cells to secure intestinal homeostasis (Figure 2).

## Group 3 ILCS in Intestinal Disease: the Other Side of the Coin

**Figure 3:**
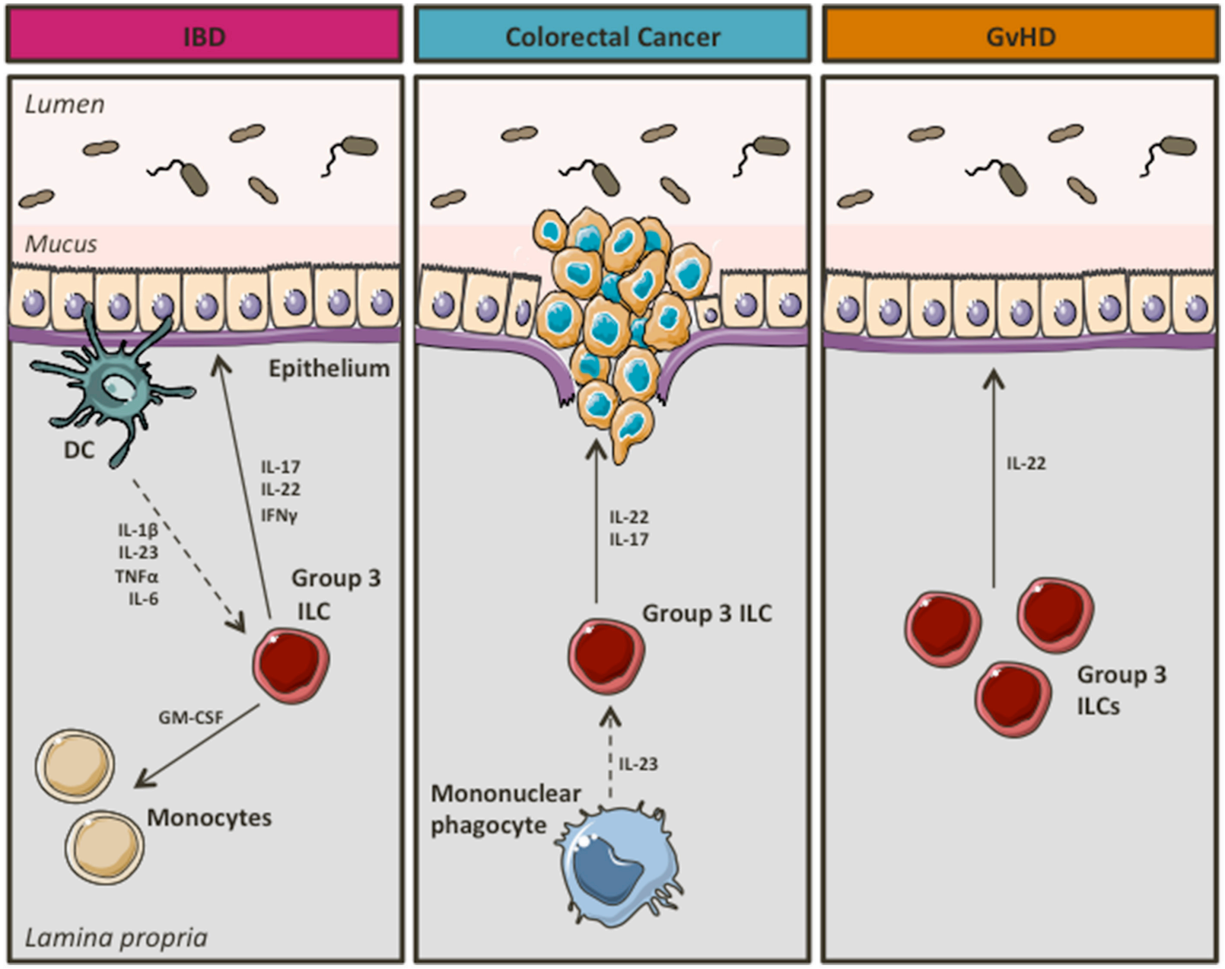
Group 3 ILCs during intestinal disease. In chronic inflammation group 3 ILCs seem to acquire a pro-inflammatory phenotype, thus contributing to the development of IBD and/or colorectal cancer. However, they appear to have a beneficial role in GvHD.

In a landmark study, Buonocore et al. showed that chronic infection of immunodeficient mice with *Helicobacter hepaticus* resulted in RORγt^+^ ILC3 mediated gut inflammation. ILC3s produced high levels of IL-17 and IFNγ in response to IL-23, thus contributing to the development of T-cell independent inflammation (26). Similarly, ILC3s were crucial for the development of innate mediated colitis using the anti-CD40 model of IBD (26). In support of a pathogenic role of ILC3s in intestinal inflammation (Figure 3), Geremia et al. showed that IL-23 responsive ILC3s were increased in the intestines of patients with CD, where in response to IL-23 they produced high levels of IBD-relevant cytokines such as IL-17 and IL-22 (66). Shedding more light into the mechanisms underlying ILC3-driven intestinal pathology, Pearson et al. showed that ILC3s use GM-CSF to attract inflammatory monocytes on site, whereas mobility of ILC3s in and out of cryptopatches appeared to induce inflammation at non-inflamed sites (67). Recently, Bauché et al. showed that Tregs were able to prevent ILC3-induced colitis (68). In particular, latent activation gene 3 (LAG3) expressing Tregs reduced IL-1β and IL-23 production by CX3CR1^+^ macrophages resulting in impaired IL-22 production by ILC3s and therefore attenuated disease (68). Moreover, using *Tbx21*^−/−^*Rag2*^−/−^ Ulcerative Colitis (TRUC) mice, which develop spontaneous colitis with clinical features that resemble aspects of human UC (69), Powell et al. showed that IL-23 and TNFα produced by CD103^−^CD11b^+^ mononuclear phagocytes activated ILC3s to drive intestinal inflammation through production of IL-17 (70). In particular, NCR^−^ ILC3s, the major ILC subset present in the colon of TRUC mice, were potent producers of IL-17 and IL-22 in response to IL-1α and IL-23, an effect that was more profound in the presence of IL-6 (71). Interestingly, *in vivo* blockade of IL-6 using neutralizing antibodies significantly attenuated colonic inflammation in TRUC mice (71).

Interestingly, group 3 ILCs have also been associated with a pro-inflammatory, pathogenic role in cancer (Figure 3), a feared complication of unopposed inflammation in patients with UC (72, 73). IL-17^+^IL-22^+^ ILCs accumulate in colorectal cancer occurring in *Helicobacter hepaticus* associated colorectal cancer. ILC depletion alleviated invasive cancer in this model (74). Importantly, IL-22, but not IL-17, was necessary for cancer maintenance (74). In support of ILC3s having a role in promoting colorectal cancer, Chan et al. showed that ILC3s were key drivers of IL-23 induced tumorigenesis, however in this setting ILC3 actions were mediated by IL-17 (75).

Intestinal inflammation can also occur as a result of graft vs. host disease (GvHD) in recipients of allogenic hematopoietic stem cell transplants (AHSCT) as a treatment of hematopoietic cell disorders including blood cancers (76). Although several studies suggest that ILC3s may contribute to intestinal inflammation promoting the development of IBD or colorectal cancer, in GvHD they might be beneficial (Figure 3). Hanash et al. showed that IL-22 production by ILC3s was increased in patients following pretransplant conditioning, whereas IL-22 levels were reduced upon emergence of GvHD (77). Notably, IL-22 deficiency in recipients resulted in significant intestinal inflammation and tissue damage (77). Moreover, Munneke et al. suggested that elevated numbers of NCR^+^ ILC3s in peripheral blood of leukemia patients following AHSCT were associated with reduced risk of GvHD (78).

## Concluding Remarks

Although they are phenotypically diverse, and exist as multiple different subsets group 3 ILCs are probably the best-characterized ILC lineage, and they appear to play an important role regulating the balance between maintenance and loss of intestinal homeostasis. A decade after their discovery, ILC3s have emerged as important regulators of inflammation at mucosal surfaces, and in the gut in particular ILC3s form a dynamic network of interactions with the microbiome, other immune cells, the intestinal epithelium and enteric neurons to secure intestinal homeostasis. However, ILC3s may also promote inflammation leading to the development of IBD and/or colorectal cancer. Additional work is needed to scrutinize ILC3 biology and their contributions to intestinal disease. Targeting ILCs, their key upstream activating mediators (e.g., IL-23, IL-1β, or IL6), their survival factors (e.g., IL-7), or their effector cytokines (IL-22, IL-17, and IFNγ) hold promise for treating inflammatory diseases such as IBD.

## Author Contributions

EP wrote the manuscript and designed the figures. NP critically reviewed the manuscript.

### Conflict of Interest Statement

The authors declare that the research was conducted in the absence of any commercial or financial relationships that could be construed as a potential conflict of interest.
